# Flame thermometry using laser-induced-grating spectroscopy of nitric oxide

**DOI:** 10.1007/s00340-018-6912-2

**Published:** 2018-02-13

**Authors:** Andrew Luers, Anna-Lena Salhlberg, Simone Hochgreb, Paul Ewart

**Affiliations:** 1 Department of Physics, Clarendon Laboratory, Oxford University, Parks Road, Oxford, OX1 3PU UK; 2 Department of Mechanical Engineering, Cambridge University, Trumpington Street, Cambridge, CB2 1PZ UK

## Abstract

A systematic study of laser-induced thermal-grating scattering (LITGS) using nitric oxide as an absorbing species is presented as a means of thermometry in air-fed combustion. The relative contributions to the scattered signal from degenerate four-wave mixing, DFWM, and from laser-induced thermal-grating scattering, LITGS, are studied in the time domain for NO in N_2_ buffer gas up to 4 bar, using a pulsed laser system to excite the (0,0) γ-bands of NO at 226.21 nm. LITGS signals from combustion-generated NO in a laminar, pre-mixed CH_4_/O_2_/N_2_ flame on an in-house constructed slot burner were used to derive temperature values as a function of O_2_ concentration and position in the flame at 1 and 2.5 bar total pressure. Temperature values consistent with the calculated adiabatic flame temperature were derived from averaged LITGS signals over 50–100 single shots at 10 Hz repetition rate in the range 1600–2400 K with a pressure-dependent uncertainty of ± 1.8% at 1 bar to ± 1.4% at 2.5 bar. Based on observed signal-to-noise ratios, the minimum detectable concentration of NO in the flame is estimated to be 80 ppm for a 5 s measurement time at 10 Hz repetition rate.

## Introduction

Temperature is a key parameter in combustion processes, since it affects the rate of many of the chemical reactions involved and the levels of product species including unwanted pollutants such as NO_*x*_. Temperature fluctuations associated with thermo-acoustic instabilities in technical combustion devices are correlated with local variations in the concentration of NO in particular. Accurate and precise measurements of temperature are important, therefore, for understanding and mitigation of these effects, for example, in gas turbine engines. NO is a product of most air-fed combustion processes and provides a medium by which the temperature of burnt gas can be found by spectroscopic measurements. Since NO is a relatively stable product, compared to radicals such as OH, it has some advantages as a target species in flames for optical and laser-based measurement techniques. Both linear and non-linear optical methods have been developed for combustion diagnostics and offer the benefits of remote, non-invasive, measurements of temperature and species concentrations in both laboratory flames and engines [1, 2]. The linear technique of laser-induced fluorescence, LIF, has been extensively used for measurements of NO in combustion situations owing to its high sensitivity and the facility it offers for imaging of concentration and temperature distributions using a planar excitation beam (planar laser-induced fluorescence, PLIF) [1–4]. The disadvantages of LIF and PLIF include the difficulty of quantitative analysis owing to unknown collisional quenching effects on the signal intensity, susceptibility to optical interference from scattering or emissions in particle-laden or highly luminous environments and the need for good optical access to collect the spontaneously radiated signal efficiently. Non-linear optical methods have some advantages owing to the laser-like property of the signal beams that provide spatial and temporal resolution, discrimination against luminous backgrounds and the ability to use limited optical access. Coherent anti-Stokes Raman Scattering, CARS, is a four-wave mixing process that has been widely used for thermometry and, in some cases, for species concentrations, but its use is generally restricted to majority species with N_2_ being the most commonly used medium [1, 5]. Resonant four-wave mixing interactions, however, provide high species and state selectivity and allow detection of species in intermediate or trace concentrations [6]. Transient species such as OH as well as stable species like NO that may be present in only trace amounts have been detected in flames using these methods. Degenerate Four-Wave Mixing, DFWM, is a non-linear process similar to CARS but, owing to the fully resonant interactions involved, it allows coherent detection of the combustion species OH in a methane/air flame [7]. Thermometry of flames using DFWM spectra of OH was also demonstrated for time-averaged point measurements [8], 2D temperature maps [9] and single-shot measurements using broadband laser excitation [10, 11]. DFWM was also used to detect NO in flames [12] and measurement of relative concentrations of NO in a firing spark-ignition engine was also demonstrated [13, 14].

The physical process leading to DFWM signal generation involves the creation of a spatially-periodic modulation of the medium’s refractive index by interaction with two interfering laser (pump) beams of the same frequency. This laser-induced grating is established by the coherence induced by the resonant interaction with the molecules and is sometimes referred to as a population grating. Scattering of a third (probe) beam, again of the same frequency, from this grating produces the fourth, or signal, beam. Collisional dephasing of the molecular coherence leads to rapid decay of the signal and so the temporal shape of the signal follows that of the excitation pulses. It was recognised early on that collisions can also quench the excited molecules, leading to energy transfer to the surrounding gas medium [15]. This rapid energy transfer results in a temperature and density perturbation with the same spatial distribution as the original interference pattern, i.e., a laser-induced thermal grating. Scattering from this grating leads to a signal referred to as laser-induced thermal-grating scattering, LITGS. The thermal-grating scatters the probe beam in a DFWM experiment in the same direction as the coherent signal generated by the population grating and contributes to the measured signal intensity [16]. LITGS signals were identified in four-wave mixing experiments on OH and NO in flames [17, 18]. Therefore, both DFWM and LITGS signals can be generated simultaneously and offer potential for simultaneous measurement of different parameters such as species concentration and temperature.

A similar density perturbation is induced by electrostriction—a non-resonant effect which leads to laser-induced electrostrictive grating scattering, LIEGS [19]. The electrostrictive contribution is usually relatively insignificant when the pump beams are resonantly absorbed. Essentially, the same processes were identified as laser-induced thermal acoustics, LITA, by Cummings as a means of measuring gas dynamic or thermodynamic properties [20, 21]. Models of these processes have been developed based on solution of linearized hydrodynamic equations governing the evolution of the induced gratings and show excellent agreement with experimental observations, where the relevant gas dynamic parameters are known or can be estimated with reasonable accuracy [16, 19–23].

The LITGS signal decays exponentially as the stationary thermal grating is dispersed by molecular diffusion. Superimposed on this exponentially decaying intensity is a decaying sinusoidal modulation induced by a standing acoustic wave resulting from two, oppositely-propagating, sound waves initiated by the sudden density perturbation that established the thermal grating. The modulation frequency, *f*_osc_, is determined by the local sound speed *c*_S_ and the grating period Λ which is determined by the wavelength of the interfering beams, *λ*, and the crossing angle, *θ*:

1 $$\Lambda =\frac{\lambda }{{2\,\sin ({\theta \mathord{\left/ {\vphantom {\theta 2}} \right. \kern-0pt} 2})}}.$$

The modulation frequency is given by

2 $${f_{{\text{osc}}}}={{{c_{\text{S}}}} \mathord{\left/ {\vphantom {{{c_{\text{S}}}} \Lambda }} \right. \kern-0pt} \Lambda }.$$

Assuming that the medium obeys the ideal gas laws, the sound speed is given by

3 $${c_{\text{S}}}={\left( {\frac{{\gamma {k_{\text{B}}}T}}{m}} \right)^{{\raise0.5ex\hbox{$\scriptstyle 1$}\kern-0.1em/\kern-0.15em\lower0.25ex\hbox{$\scriptstyle 2$}}}},$$

where *γ* is the ratio of specific heats at constant volume and pressure, *m* is the mean molecular mass and *k*_B_ is Boltzmann’s constant. Hence, from the measured oscillation frequency, *f*_osc_, the temperature is derived from

4 $$T=\frac{m}{\gamma }\frac{{{\Lambda ^2}}}{{{k_{\text{B}}}}}f_{{{\text{osc}}}}^{2}.$$

It was recognised that analysis of LITGS or LITA signals would thus provide a means of measuring the temperature [20]. The distinguishing feature of LITGS and LITA for thermometry is that the temperature is derived from a measured frequency rather that a relative intensity or spectral intensity profile as in other laser-based methods. Cummings was able to derive the temperature of laboratory air from time-averaged LITA signals arising from absorption by trace amounts of NO_2_ [20]. The first measurements of flame temperature using LITGS were reported by Latzel et al. using OH in a high-pressure methane/air flame [24]. More recently, the high precision available from LITGS thermometry, on the order of 0.1%, has led to increased interest in the technique [25]. In-cylinder temperatures of a firing SI engine were measured with high single-shot precision by Williams et al. using the fourth harmonic of an Nd:YAG laser at 266 nm, to produce the grating in fuel vapour [26]. Hell et al. have used the fundamental output of an Nd:YAG laser at 1064 nm as the pump beams for LITA with a pulsed probe beam, to measure temperatures in methane/air and H_2_/air flames as well as a hot supersonic H_2_/air free jet [27]. Finally, Sahlberg et al. have demonstrated LITGS thermometry of flames using mid-IR pumps around 3000 nm and a cw probe laser at 457 nm to generate LITGS signals from combustion-generated H_2_O [28].

In this paper, we present a limited, but systematic, study of LITGS thermometry of flames using combustion-generated NO. As noted above, the interest in NO stems from its ubiquitous presence in air-fed combustion and its importance as an atmospheric pollutant. The ability to use NO as an absorber for product temperature measurements would enable the LITGS technique to measure the temperature uniformity around the nozzle guide vanes, an important measure for gas turbine performance [29, 30]. Furthermore, it would create the ability to directly measure the amplitude of temperature fluctuations, and thus detect the emergence of localised regions of elevated temperature, called entropy spots, as a source of pressure fluctuations [31–33]. As a relatively stable species, NO provides a useful target molecule with which to monitor temperature in the burned-gas zones of flames and technical combustion devices. In principle, the strength of the signal gives a measure of the concentration of the NO, the production of which is highly temperature-dependent. The measurement of both temperature and concentration is, therefore, attractive both for understanding instabilities as well as NO-emission production.

The main focus of the present work is to develop thermometry using LITGS in combustion-generated NO. As a precursor to the experiments to measure temperature, a semi-quantitative experimental analysis of the relative roles of DFWM and LITGS was conducted. The results illustrate the competing effects of DFWM and LITGS processes in the time domain. In what follows we outline the experimental procedure for LITGS thermometry and describe calibration measurements to improve measurement accuracy and to determine the inherent single-shot precision and uncertainty of measurements using time-averaged signals. Temperature measurements are then reported in a laminar, pre-mixed CH_4_/O_2_/N_2_ flame as a function of pressure, oxygen content and position in the flame. Finally, some observations are made regarding concentration measurements and the minimum concentration detection limit of NO in the flame using LITGS.

## Experimental arrangement and method

**Fig. 1:**
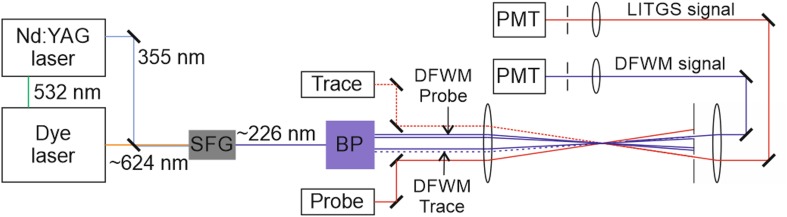
Experimental arrangement for simultaneous observation of DFWM and LITGS signals. The output of the dye laser at 624 nm is mixed with the third harmonic of the Nd:YAG laser at 355 nm in a sum frequency generation (SFG) crystal to produce light at 226 nm for DFWM pump and probe pulses producing the pulsed DFWM signal recorded on a photomultiplier tube (PMT). BP is a system of two-thick beam splitting plates that divide the 226 nm beam into four parallel beams. The probe is a cw diode-pumped solid-state laser at 671 nm to read out the LITGS signal recorded on a separate PMT

The experimental arrangement for simultaneous observation of both DFWM and LITGS is shown in Fig. 1. The second harmonic and third harmonic outputs of a single-mode pulsed Nd:YAG laser (Continuum Powerlite 9000) are used to pump a dye laser and for sum–frequency mixing with the dye laser output, respectively. In all the thermometry experiments, the 226 nm radiation to excite the (0,0) γ-bands of NO was generated by sum frequency mixing the output of the dye laser at 624 nm with the third harmonic of the pump laser at 355 nm. The dye laser used a modeless laser system to produce 10 mJ pulses with a bandwidth variable down to 0.3 cm^− 1^ [34]. Sum–frequency generation in a BBO crystal produced about 1 mJ at 226.12 nm in a 5 ns pulse. The wavelength was inferred from measurements of the wavelength of the Nd:YAG harmonic outputs and of the dye laser using a pulsed wavemeter (BurleighWA-4500). This ultraviolet beam was split into four parallel beams by a system of thick beam splitter plates, indicated as BP in Fig. 1, similar to that used in previous experiments and provided stable beam alignment for both DFWM and LITGS experiments [13, 35]. For DFWM, two of the four beams provided the pumps in a forward folded BOXCARS arrangement when intersected by use of a 750 mm focal length crossing lens [36]. This geometrical arrangement defined an interaction region of length 35 mm and width 1.5 mm. A third beam was used as the probe beam and the fourth beam provided a tracer beam to mark the path of the signal beam. The tracer beam was blocked during the DFWM generation process, but it allowed accurate alignment of the signal beam onto a photomultiplier tube, PMT, to record the signals (Hamamatsu H10721-20).

The grating generated by the two pump beams for DFWM also provided the excitation for the thermal grating which was probed by a continuous wave (cw) non-resonant probe beam incident at the Bragg angle appropriate for its wavelength and the grating spacing Λ. The probe laser for this LITGS process was a diode-pumped solid-state cw laser emitting 600 mW of power at 671 nm in a bandwidth of approximately 0.07 cm^− 1^ (CNI model MLL-FN-671-1W). An HeNe laser was used as a tracer beam as an aid to direct the signal beam onto a separate photomultiplier tube (Hamamatsu H10721-20). A red-transmitting filter was placed in the signal path to shield this PMT from scattered laser light at the pump wavelengths. Alignment of the DFWM and LITGS beams was facilitated by use of the parallel-plate beam splitters for the pump beams and alignment of the probe and tracer beams was aided using a system of masks with apertures to define the position and hence crossing angle of each beam in the interaction region [13, 35].

A stainless steel cell and gas handling system was used to provide NO at selected partial pressures in a buffer gas of N_2_. Total pressures could then be set in the range of 10 mbar to 4 bar. This cell was placed in the interaction region for generation of both DFWM and LITGS signals. Alternatively, the cell was replaced in the interaction region by a burner that could be operated at pressures up to 3 bar. The burner itself was an in-house constructed slot burner of width 0.5 mm and length 40 mm with the long axis aligned parallel to the symmetry axis of the incoming pump laser beams. This provided a flame region of approximately constant temperature that was longer than the interaction region defined by the intersecting pump beams. This arrangement avoided the problem of the relatively long measurement region containing regions of differing temperature. Mass flow controllers were used to adjust the flow of methane, oxygen, and nitrogen to a mixing chamber to establish a pre-mixed laminar flame on the burner. The burner position within the pressure chamber could be adjusted relative to the interaction region defined by the crossing beams which remained fixed thus allowing measurements as a function of the height above burner, HAB.

The accuracy of temperature values derived using Eq. (4) depends, inter-alia, upon having an accurate measurement of the grating spacing, Λ. A measure of Λ can be derived from the dimensions of the geometrical arrangement, but a more accurate value is obtained by calibration measurements at a known temperature and gas composition using Eq. (4). Calibration measurements were carried out using the cell at temperatures around ambient, but with elevated pressure to increase the accuracy in measuring the acoustic modulation frequency, *f*_osc_ [25]. The cell was filled with a gas mixture containing NO at a partial pressure of ~ 5 mbar and up to 3 bar of N_2_. The cell temperature could be adjusted over a small range around ambient and was monitored using a calibrated K-type thermocouple (Tenma). The shot-to-shot variation in *f*_osc_ (or derived temperature) for measurements in the cell gives a measure of the single-shot precision of the technique for measurements at a given temperature and pressure. The value of *f*_osc_ is found by analysis of the temporal behaviour of the LITGS signal. The signal-to-noise ratio for the cell-based measurements at ambient temperatures and pressures in the range 1–3 bar was of sufficiently high quality to allow accurate determination of *f*_osc_ by a Fourier transform of the signal. For weaker signals obtained at flame temperatures or lower pressures, more accurate values of *f*_osc_ were found by finding a best-fit theoretical signal to the data and finding the oscillation frequency of this model signal. This approach has the effect of minimizing the deleterious effect of noise on determining the frequency by the Fourier transform method.

## Results and analysis

### LITGS contributions to DFWM signals

Danehy et al. made an extensive study of the extent to which LIGS signals from thermal gratings contributed to measured signal intensities in DFWM experiments in NO and OH [18]. They observed that thermal gratings dominated the signal generation in high-density (i.e., cold, high pressure) environments, whereas population gratings, determined by the molecular coherence, dominated in low-density (i.e., hot, low-pressure) environments. At intermediate conditions, it was found that the LITGS and DFWM signals could be of comparable magnitude. The relative contributions of the two processes were studied by variation of the collisional quenching rate responsible for energy transfer to the thermal grating using different mixtures of the buffer gases, N_2_ and CO_2_, which have quenching rates for NO differing by several orders of magnitude. They also noted that the temporal behaviour of the two processes was different. The coherent DFWM process decayed rapidly by collisional dephasing, leading to a signal that followed closely the time duration of the pump and probe pulses, whereas the LITGS signal from the thermal grating decayed on a much longer timescale determined by molecular diffusion and viscous damping. These studies were conducted using a pulsed probe laser, in which the time evolution of the LITGS signals was mapped by stepping the delay of the probe pulse relative to the excitation pump pulses. They noted that the LITGS signal could be completely distinguished from the DFWM signal simply by delaying the probe pulse until after the pump pulses had terminated, at which time the molecular coherence had decayed to zero. Fantoni et al. made a preliminary study of the pressure dependence of the two processes using a simple, phenomenological, model which, whilst capable of simulating the qualitative behaviour when one or other process dominated, was unable to adequately treat the situations, where the signals were comparable [37].

In the present work, we illustrate the transition from population-dominated (DFWM) signals to thermal-grating-dominated (LITGS) signals by observing the time behaviour of the four-wave mixing signal produced by a degenerate probe. Second, we show directly the competition between the two processes when the thermal grating is monitored by the non-resonant cw probe incident at the Bragg angle.

**Fig. 2:**
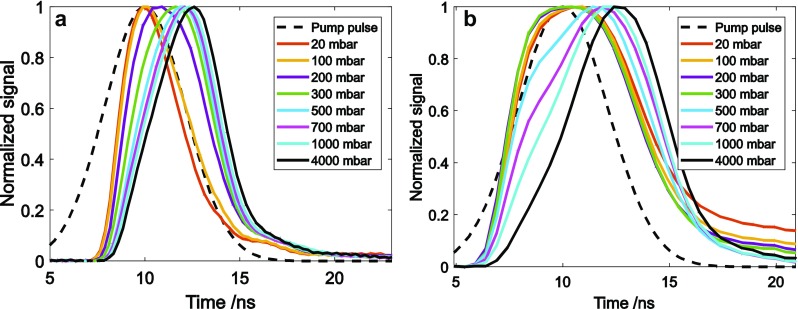
Time profile of DFWM signals in NO recorded with a pulsed probe simultaneous with the pump pulses. **a** Unsaturated and **b** saturated signals for varying total pressure of N_2_. The temporal profile of the incident pump and probe pulse is shown (dashed line, see text for discussion

Figure 2a shows the DFWM normalised signals produced by unsaturating pump beams in 20 mbar NO in the cell with varying pressure of N_2_ buffer gas to give total pressures in the range 20–4000 mbar. At the lowest pressures, below 200 mbar, the signal is contemporaneous with the pump pulses. Since the signal is proportional to the third power of the incident laser intensity the signal duration is reduced compared to that of the incident laser pulses. As the pressure increases, the peak of the signal pulse moves to later times and the shape becomes asymmetric, characterised by a relatively slow rise and fast decay. This behaviour is explained by the reduction of the coherent population-grating contribution with increasing pressure. The thermal-grating signal, however, grows during the pulse at a rate determined by the collisional quenching, but is truncated by the falling intensity of the probe pulse. The data show that above about 500 mbar N_2_ pressure, the signal is dominated by the thermal-grating contribution. Increasing the quenching rate beyond this level results in no significant additional effect on the resulting signal.

The situation is modified when the pump intensities are sufficient to saturate the excitation of the molecular coherence. With the values of the parameters used in the present experiments (laser linewidth, beam area, transition linewidth and at 1 bar pressure), an upper bound of the saturation pulse energy for the transition being pumped is estimated to be ~ 650 mJ [38]. Figure 2b shows the normalized signal for saturating pump intensities from which it is seen that at lower pressures, saturation leads to a broadening of the signal pulse in time and the effects of collisions are partially compensated by the higher pumping rate. Consequently, higher pressures are required to cause the thermal grating to dominate the coherent population contribution. A significant contribution from DFWM is apparent at pressures of 500–700 mbar in the saturated case, Fig. 2b, relative to the unsaturated case, Fig. 2a.

**Fig. 3:**
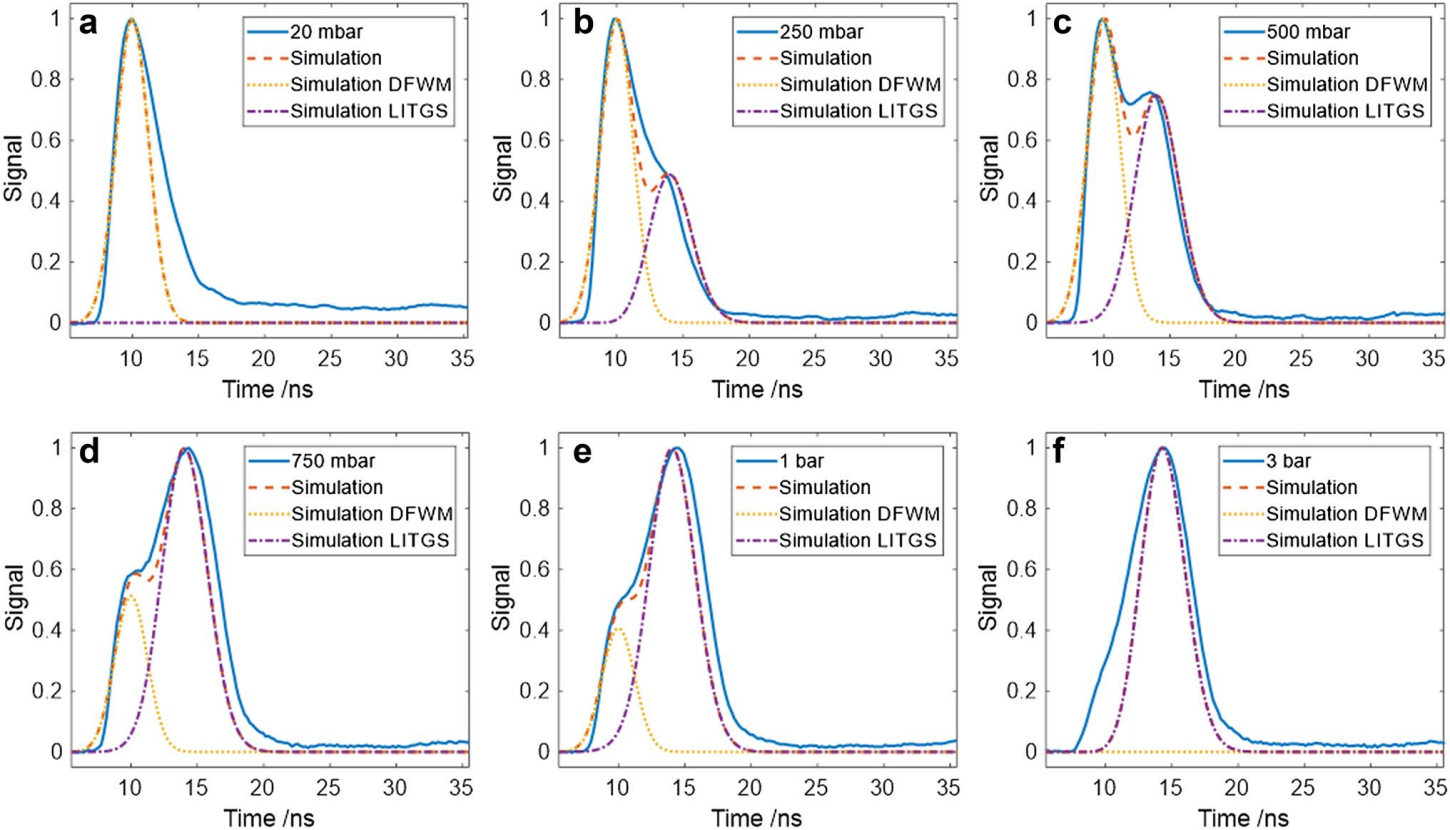
Experimental time profiles of DFWM signals in NO (20 mbar) with increasing pressure of N_2_ (solid blue line). Simulated signals (dashed red line) are the result of the addition of a simulated DFWM contribution (dashed yellow line) and a simulated LITGS signal (dashed purple line). Note that at the lowest pressure, 20 mbar, the LITGS component is negligible, and at the highest pressure, 3 bar, the DFWM component is relatively insignificant, see text for details

The transition from the DFWM (population-dominated) signal to one dominated by LITGS (thermal-grating-dominated) is illustrated in Fig. 3 for 20 mbar NO with N_2_ pressures in the range 20 mbar–3 bar. The temporal behaviour of the signal generated by the pulsed degenerate probe shows the effect of both grating contributions. The signal profile was modelled heuristically by combining contributions from DFWM and LITGS in the time domain. The DFWM signal was modelled using the approach of Abrams and Lind and the LITGS signal was simulated using the model of Paul et al. [16, 39]. The relative signal strengths were adjusted to obtain the best fit to the experimental data as shown by the red dotted line relative to the experimental profiles indicated by the blue solid line. The onset of the LITGS contribution is evident in the growth of the second peak at around 15 ns delay relative to the origin.

**Fig. 4:**
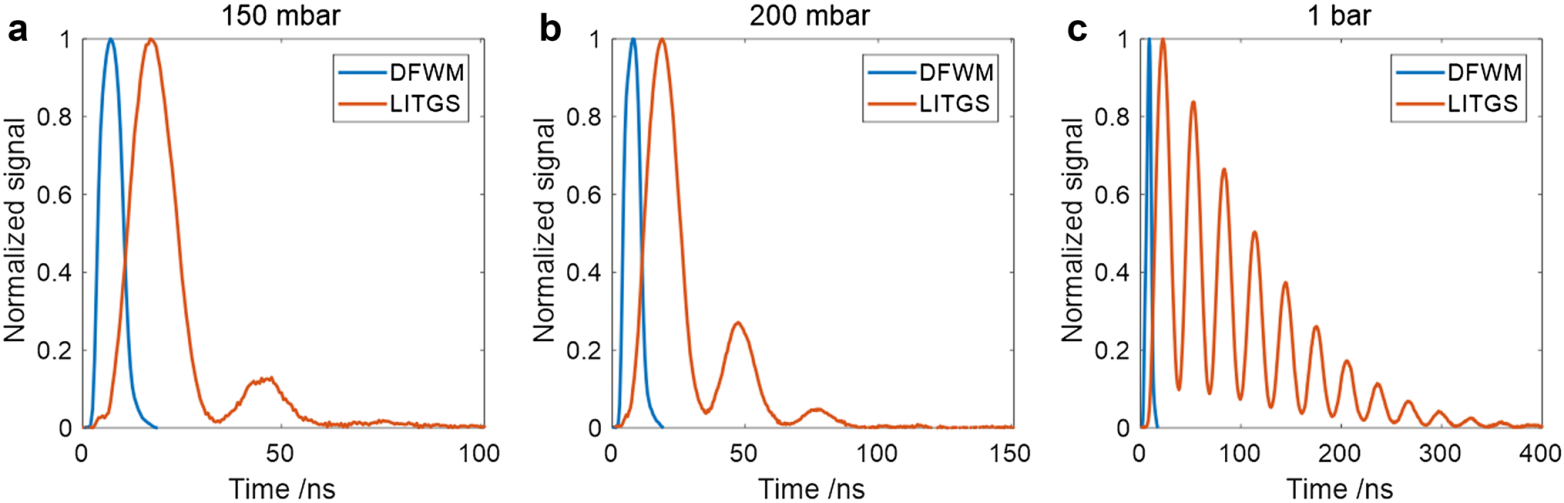
DFWM and LITGS signals recorded simultaneously for increasing pressure of N_2_ buffer gas at **a** 100 mbar, **b** 200 mbar and **c** 1 bar. The data are averages over 100 single shots. The blue line shows the DFWM signal produced by a pulse intensity approximately equal to the saturation value at 1 bar

The LITGS contribution can also be detected using a non-degenerate, cw probe beam which shows the long-term behaviour of the LITGS signal. Simultaneous records of the pulsed DFWM signal and of the LITGS signal generated by the cw probe at 671 nm incident at the appropriate Bragg angle are shown in Fig. 4 for varying pressures of N_2_ between 100 mbar and 1 bar. Above 200 mbar, the oscillatory behaviour of the LITGS signal becomes sufficiently apparent to provide increasingly precise temperature measurement as the pressure is increased. The timing of the signals is measured relative to the incident pump pulses and the effectively instantaneous DFWM signal generated at the lowest pressure. The increasing delay of the pulsed signal peak relative to this time, with increasing pressure, is correlated with the increasing domination by the thermal-grating contribution, as shown in Fig. 3.

**Fig. 5:**
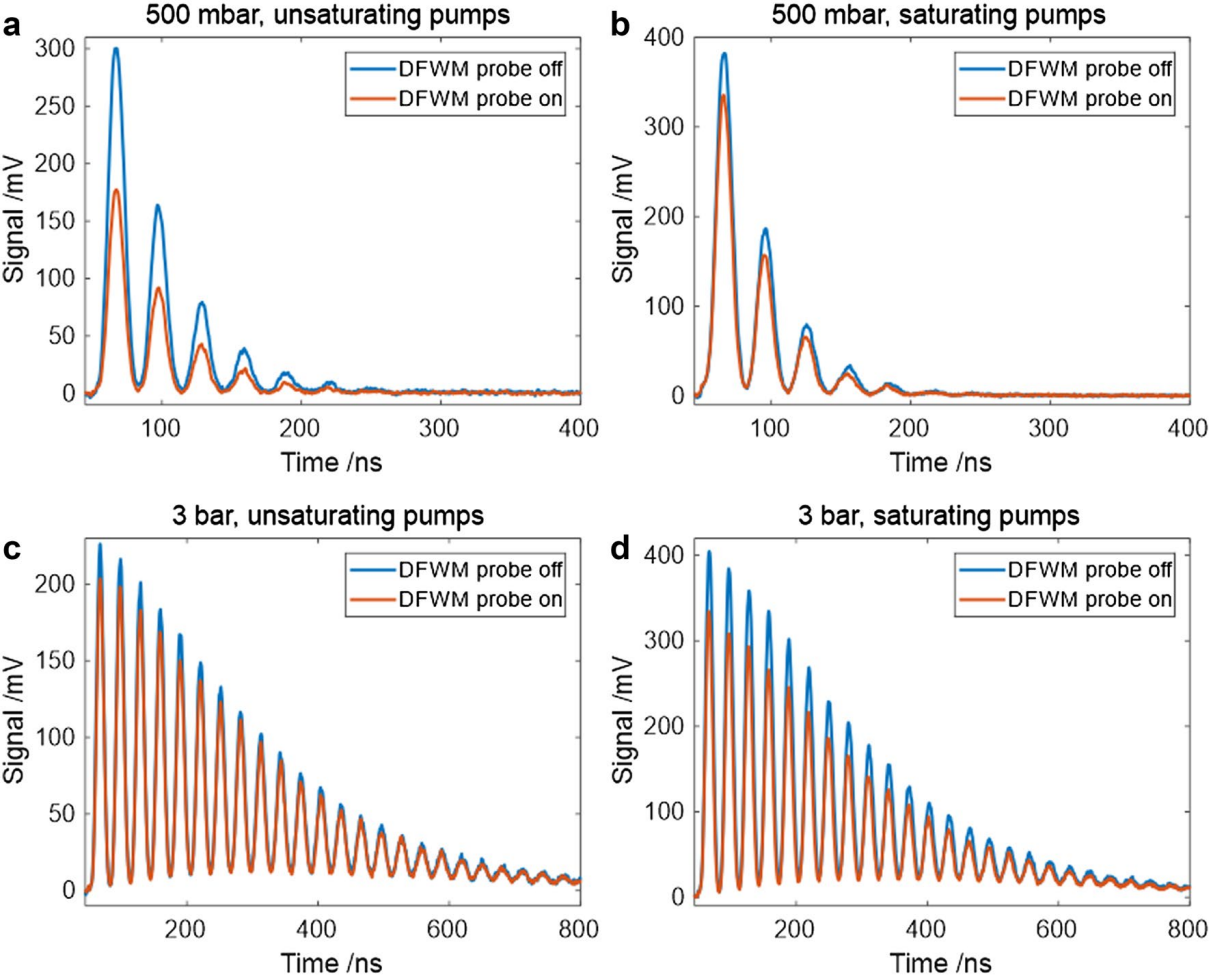
Simultaneously recorded DFWM and LITGS averaged signals showing the effect of the degenerate probe used for DFWM on the magnitude of the LITGS signals. The blue lines show the LITGS signal in the absence of the degenerate probe used for DFWM and the red line the signal in the presence of the degenerate probe. **a, b** Show unsaturated and saturated pump conditions at 500 mbar. **c, d** Show the effect of the DFWM probe at 3 bar where the observed difference in the LITGS signal is not statistically significant

When the degenerate probe pulse at 226 nm was incident on the induced grating at the same time as the LITGS probe beam at 671 nm, the intensity of the LITGS signal was observed to decrease, as shown in Fig. 5 especially in the case of unsaturating pump fields, as shown in Fig. 5a, b. At a total pressure of 500 mbar with unsaturating pump and probe intensity, the addition of the DFWM probe reduces the LITGS signal intensity by a factor of 1.7. When saturating pumps and probe are used the reduction factor is 1.1. In the unsaturated case, the DFWM probe is relatively more effective in competing with collisional relaxation, thus reducing the amount of energy available for transfer to the thermal grating. Under saturating pump conditions, the excited state population is more effectively re-pumped thus allowing more energy to be transferred to the thermal grating.

At the higher pressure of 3 bar, the difference in the LITGS signal intensity induced by the DFWM probe for unsaturated and saturated conditions is 1.1 and 1.2, respectively. Measurement errors of the intensity of each signal are estimated to be up to 5%, and therefore, a relative change of 10% is of the same order of magnitude. Consequently, the difference between saturated and unsaturated signals at the higher pressure of 3 bar; Fig. 3c, d shows not statistically significant. At these higher pressures, the population dynamics are dominated by the higher collision rate; the DFWM signal is reduced effectively to zero (see Fig. 3f) and the de-excitation by the degenerate DFWM probe is relatively insignificant. Furthermore, at the higher pressure, the intensity required to saturate is higher, so when the same intensity is used at 500 mbar and 3 bar, the saturation effect is reduced at the higher pressure relative to the unsaturated case.

### LITGS thermometry using NO

The accuracy and precision of measurements depend on evaluation of systematic errors and random fluctuations associated with variations in the measurement region and of the optical system. The accuracy of the temperature value derived from the LITGS signal depends upon having an accurate measure of the grating spacing Λ and an accurate value for *γ*/*m* which in turn depends on having an accurate estimation of the flame composition. The flame composition was derived from flame equilibrium models in the literature—specifically, the concentrations of the various species present in a CH_4_/O_2_/N_2_ flame were based on a chemical equilibrium calculation as a function of temperature using the NASA-CEA computer program of Gordon and McBride [40]. This composition was used to calculate a weighted mean value of *γ*/*m* for an adiabatic flame temperature and this value was used to derive the flame temperature from the measured value of *f*_osc_ for the LITGS signal in the flame. It was found that the difference in *γ*/*m* over the range covering the adiabatic flame temperature and the values derived from the LITGS measurements was insignificant relative to other experimental uncertainties. Consequently, the value of *γ*/*m* for adiabatic flames was used in the derivation of temperature in all subsequent measurements. The most accurate method to determine Λ is by use of calibration under known conditions and this is described in the following section. Estimates of the single-shot precision inherent in the technique were obtained from the standard deviations of batches of up to 100 single-shot measurements in a stable cell environment. Flame flicker and small fluctuations in gas flow contribute to shot-to-shot variations in the flame measurements. However, the flame to be investigated was a stable laminar flow, and so, a degree of averaging over the flame fluctuations was achieved by deriving temperatures from an averaged signal of 100 single shots.

#### Calibration and data analysis

**Fig. 6:**
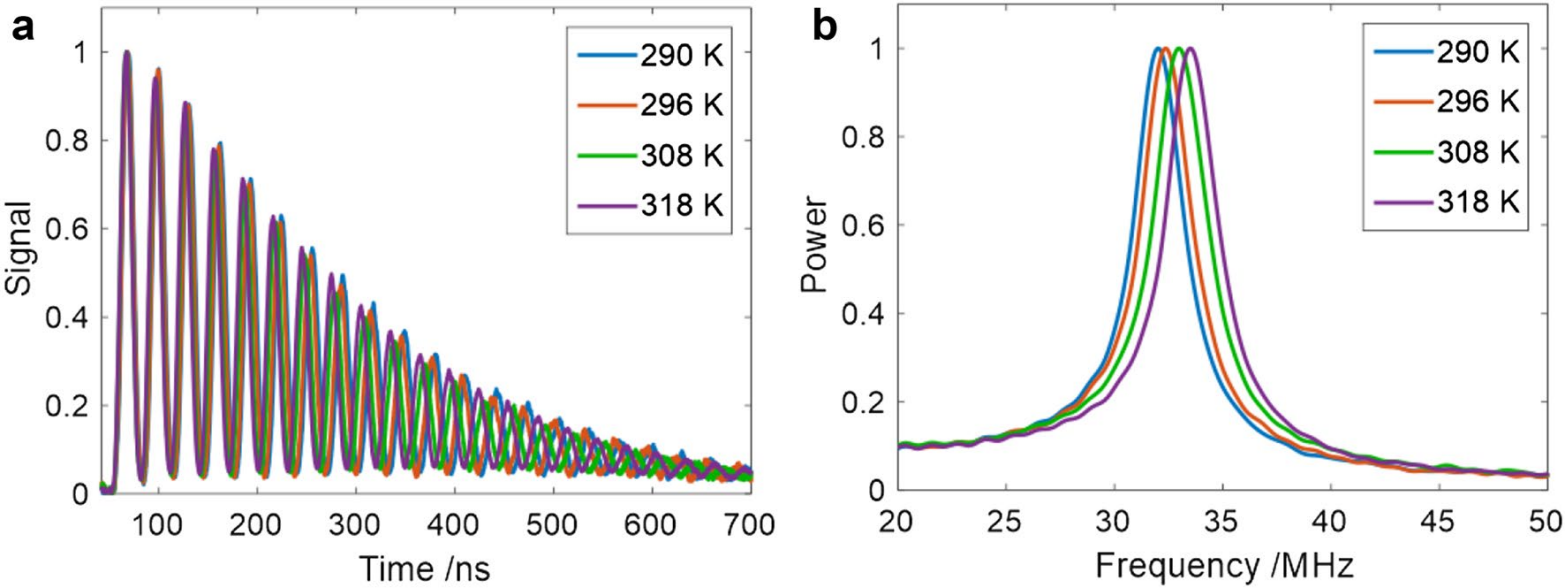
Calibration measurements in test cell with 20 mbar NO and 3 bar N_2_, **a** 50-shot averaged LITGS signals at temperatures between 290 and 320 K. **b** Fourier transform of signal in **a** showing good resolution of the fundamental acoustic frequency *f*_osc_

Using the experimental procedure outlined above, the grating spacing, Λ, was determined by calibration measurements in a cell at 3 bar total pressure. Figure 6a shows averaged LITGS signals over 100 single shots in the cell at four different cell temperatures. The values of the acoustic modulation frequency, *f*_osc_, are readily and accurately determined from the Fourier transforms, as shown in Fig. 6b. From such calibration measurements, the value of Λ used in the subsequent flame thermometry was determined to be 20 ± 0.05 µm.

A measure of the inherent single-shot precision of the technique was obtained from the standard deviation of the measurements over a batch of 100 single shots at room temperature, 294 K. The signal-to-noise ratio of these single-shot measurements at 3 bar was sufficiently high to allow reliable values of *f*_osc_ to be derived directly from the Fourier transforms of each signal yielding a set of temperature values with a standard deviation of ± 0.9 K, or 0.3%.

An alternative approach to deriving the temperature is to fit a model signal to the data using the temperature as the fit variable. Model signals were calculated using the approach of Paul et al. including two quenching rates to characterise the collisional energy transfer to the thermal grating [16, 25]. A non-linear least squares fitting routine in MATLAB^®^ was used to obtain the best fit to the averaged data over 100 shots. The uncertainty in the temperature derived from the goodness of fit in this way is defined as the 95% confidence interval for the non-linear least square parameter estimates obtained from the fitting routine. Using this approach the uncertainty in the derived average temperature in the cell measurements at 3 bar was ± 0.12 K or 0.04%. If the cell temperature is assumed to be stable and constant over the 10 s measurement time, then this result indicates that fits to an averaged signal provide greater precision in the derived temperature.

**Fig. 7:**
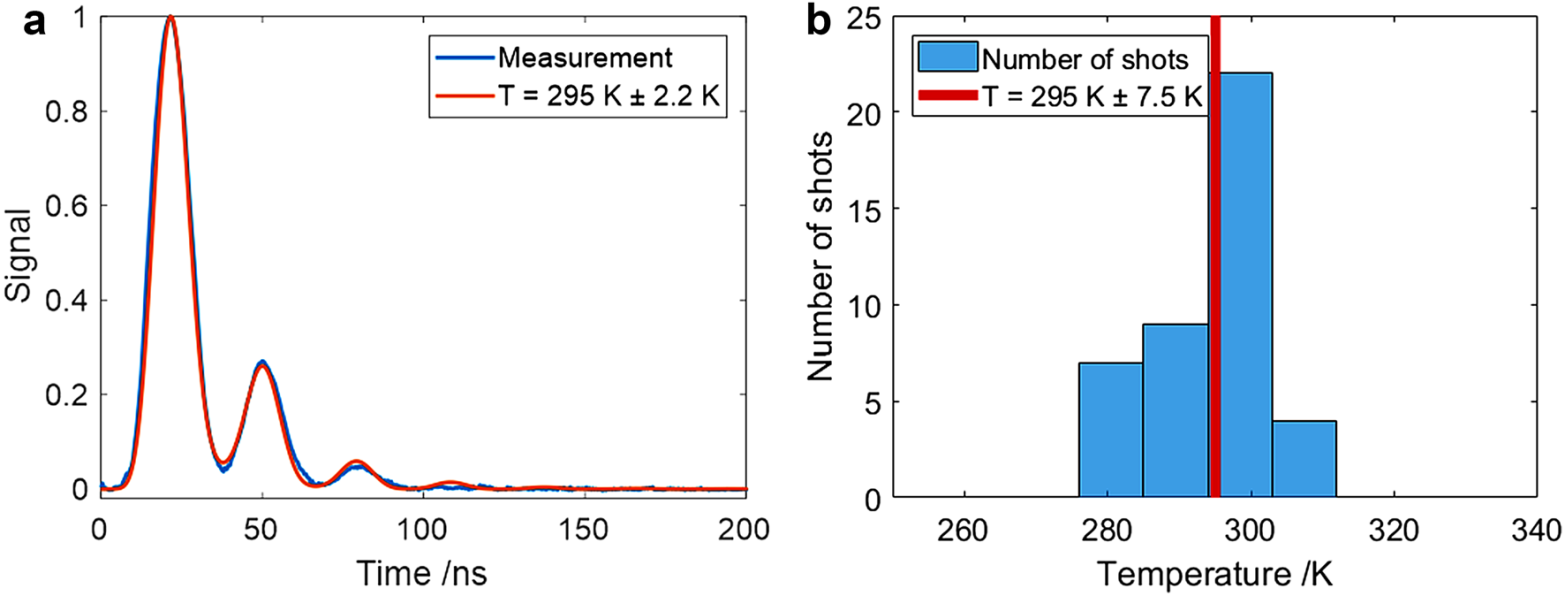
LITGS data from cell measurements at lower pressure to simulate the shorter duration signals occurring at higher temperatures. **a** 100-shot averaged LITGS signals with best-fit model signal at 200 mbar total pressure and room temperature (295 K) **b** histogram of single shots used to find average signal in **a**. This signal duration is more typical of signals at flame temperatures and atmospheric pressure

However, these calibration measurements at room temperature provide relatively long-lived signals, whereas at flame temperatures, the more rapid molecular diffusion results in much shorter duration signals with a consequent reduction in the precision with which the oscillation frequency can be determined. A measure of precision under flame conditions was estimated by reducing the cell pressure to 200 mbar at room temperature to give an LITGS signal having a comparable duration to that at flame temperatures at 1 bar. Figure 7a shows the average of 100 single shots under these conditions and the resulting single-shot precision as indicated by the histogram of temperature values derived from each of the single shots in Fig. 7b is ± 7.5 K or 2.5%. The “goodness of fit” of the best-fit model signal to the averaged signal, shown in Fig. 7a, contributed an uncertainty of ± 0.9 K. The calibration error due to uncertainty in the thermocouple measurement (± 1 K) plus the estimated error in *γ*/*m* (± 0.6 K) leads to an estimated total error of 2.2 K or 0.7%.

The measurements in the flame relied upon averaged signals over typically 50 single shots and finding the value of *f*_osc_ for the best-fit model signal. In this way the derived temperatures were averaged over the random fluctuations arising from flame flicker etc. The error arising from the fitting algorithm is in addition to other errors arising from uncertainties in the value of Λ and *γ*/*m* and fluctuations in the flame as a result of instabilities in the gas flows and flame flicker. Errors in *γ* and *m* were estimated from uncertainties in the gas flow measurements and gas composition in the flame and added in quadrature. The quoted experimental errors are, therefore, the cumulative errors arising from measurement errors and uncertainty in the fitting to the data.

The single-shot measurements in the flame had a smaller signal-to-noise ratio than the averaged signals. To improve the precision of the temperatures derived from the single-shot measurements a model signal was fitted to each single shot and the value of *f*_osc_ was obtained for the best-fit model signal. This approach reduced the deleterious effect of the noise on which otherwise produced too much uncertainty in the Fourier transform of the raw data.

### Flame measurements

**Fig. 8:**
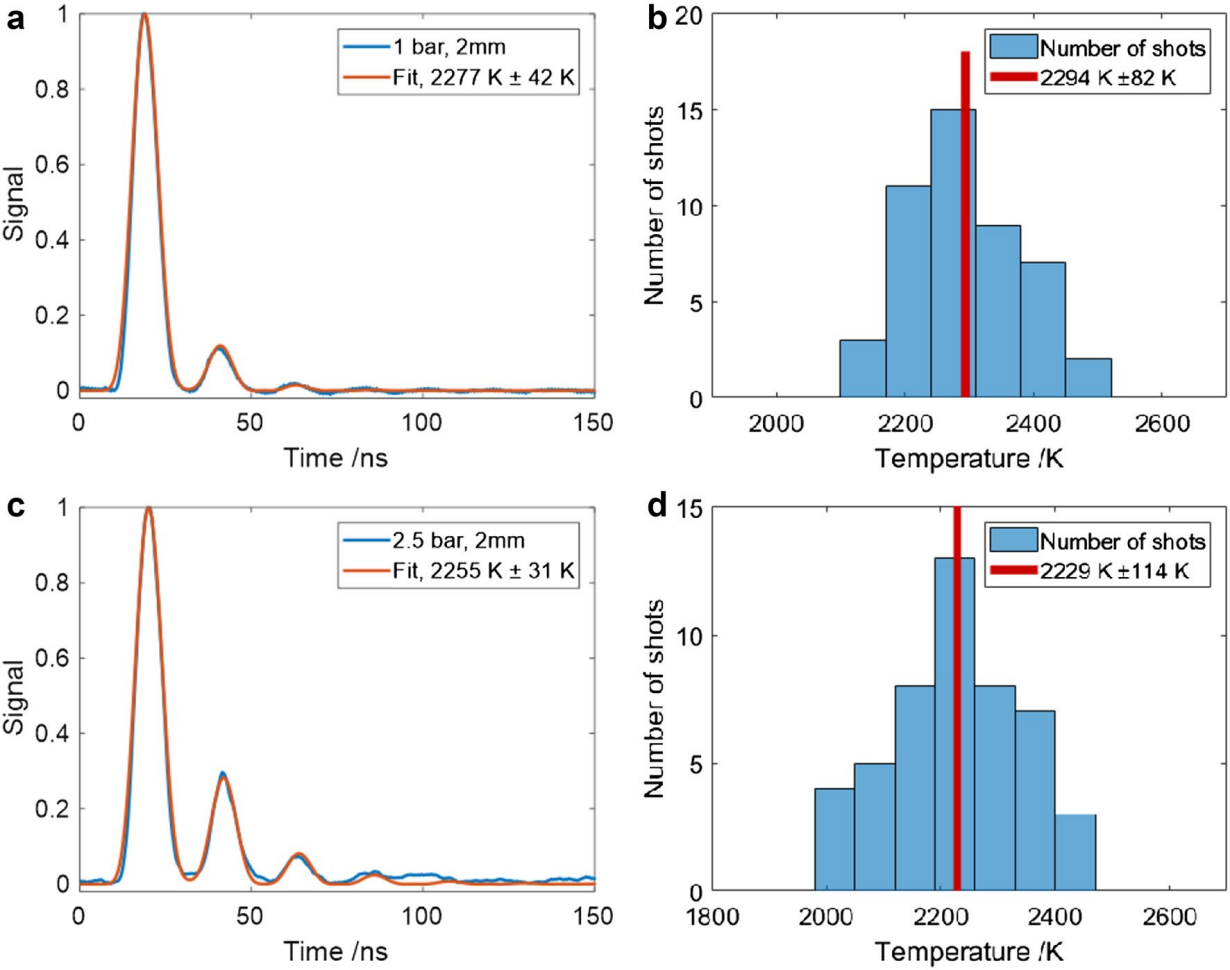
**a** LITGS signal (average of 50 single shots) from flame at 2 mm HAB and 1 bar. The red curve shows the fitted simulated LITGS signal with temperature derived from the best fit to the data. The error reflects the confidence interval of the fitted temperature, as well as the uncertainty in *γ*/*m* and Λ. **b** Temperature values from fits to 50 single-shot signals at 1 bar. The error in this case shows the standard deviation of the single-shot temperatures. **c** LITGS signal (average of 50 single shots) from flame at 2 mm HAB and 2.5 bar. The red curve shows the fitted simulated LITGS signal with temperature derived from the oscillation frequency of the best-fit model signal. **d** Temperature values from fits to 50 single-shot signals at 2.5 bar

LITGS measurements in flames are illustrated in Fig. 8 which shows data recorded at 2 mm HAB in a stoichiometric flame at pressures of 1 and 2.5 bar with O_2_:N_2_ mass ratio of 25:75. The signals shown are averaged over 50 single shots and the derived temperatures are obtained from the best-fit model to the averaged signal, as described above. In the case of the measurement at 1 bar, Fig. 8a, the error of ± 42 K on the value 2277 K consists of an uncertainty in the value of Λ of ± 10 K and an error in *γ*/*m* of ± 20 K associated with uncertainty in the mass flow measurements determining the gas composition. The remaining contribution to the error arises from the “goodness of fit” to the averaged data having a value of ~ ± 20 K for the signals at 1 bar and ~ ± 9 K for the signals at 2.5 bar. The fitting of the modelled signal to the data is confined to the first 100 ns of the signal, effectively weighting the fit, such that it is not adversely affected by the region where the signal level falls to that of the background noise. The temperature in the flame at 2.5 bar, Fig. 8c, was determined to be 2255 ± 31 K—an experimental uncertainty of ± 1.4%.

The histograms give an indication of the variation in temperatures derived from single shots. In the case of the flame at 1 bar, the value derived from the averaged signal is 2277 ± 42 K, whereas the mean of the single-shot values is 2294 ± 82 K. For the data at 2.5 bar, the respective figures are 2255 ± 31 K for the averaged data and 2229 ± 114 K for the mean of the single shots. The larger uncertainty in the mean of the single-shot data relative to that of the averaged signal indicates the effect of real fluctuations arising from flame flicker. The slight reduction in the temperature at 2.5 bar compared to that at 1 bar is explained by the reduced height of the flame at the higher pressure. This results in the measurement region being further from the flame front which sits closer to the burner with consequent increase in heat loss to the burner surface. The larger uncertainty at the higher pressure is also explained by a higher temperature gradient and the effect of flame flicker.

The temperature of pre-mixed flames is strongly affected by the oxygen content of the mixture and this, in turn, affects the concentration of NO produced by temperature-dependent reactions. Temperatures were derived from LITGS measurements in the flame at 1 bar at 2 mm HAB for differing O_2_:N_2_ ratios in stoichiometric flames as listed in Table 1.

**Table 1:** Flow rates for differing values of O_2_:N_2_ ratio in stoichiometric CH_4_:O_2_:N_2_ flames

O_2_/N_2_ ratio	CH_4_ flow (ml/min)	O_2_ flow (l/min)	N_2_ flow (l/min)	Total flow (l/min)
20/80	150	0.3	1.02	1.47
25/75	200	0.4	1.2	1.8
30/70	250	0.5	1.17	1.92
35/75	300	0.6	1.11	2
40/60	350	0.7	1.05	2.1

**Fig. 9:**
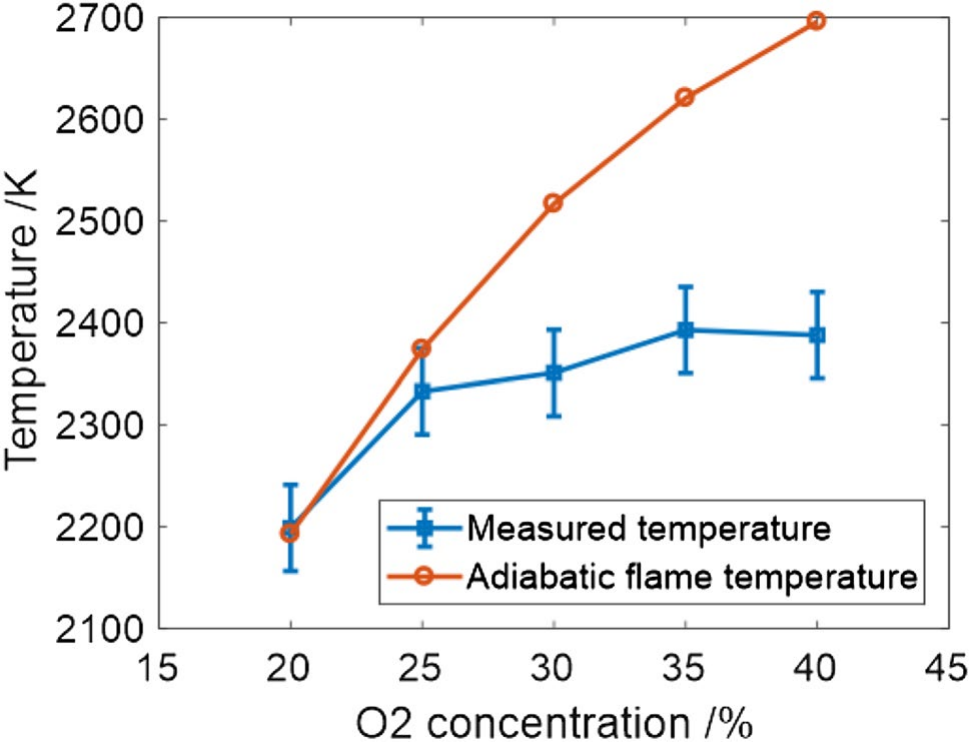
Flame temperature at 2 mm HAB as a function of oxygen content. The error bars indicate the total uncertainty based on the “goodness of fit” to averaged signals from 50 single shots and uncertainties in *γ*/*m* and Λ

The temperature values are plotted as a function of O_2_ content in Fig. 9 with a plot of the calculated adiabatic flame temperature for these gas mixtures. The deviation from the adiabatic temperature is explained largely by heat loss to the burner surface. This heat loss is increased at higher pressures as the flame sits closer to the surface. Additionally, since the measurement point remains at the same HAB, the smaller flame results in measurement further away from the flame front.

**Fig. 10:**
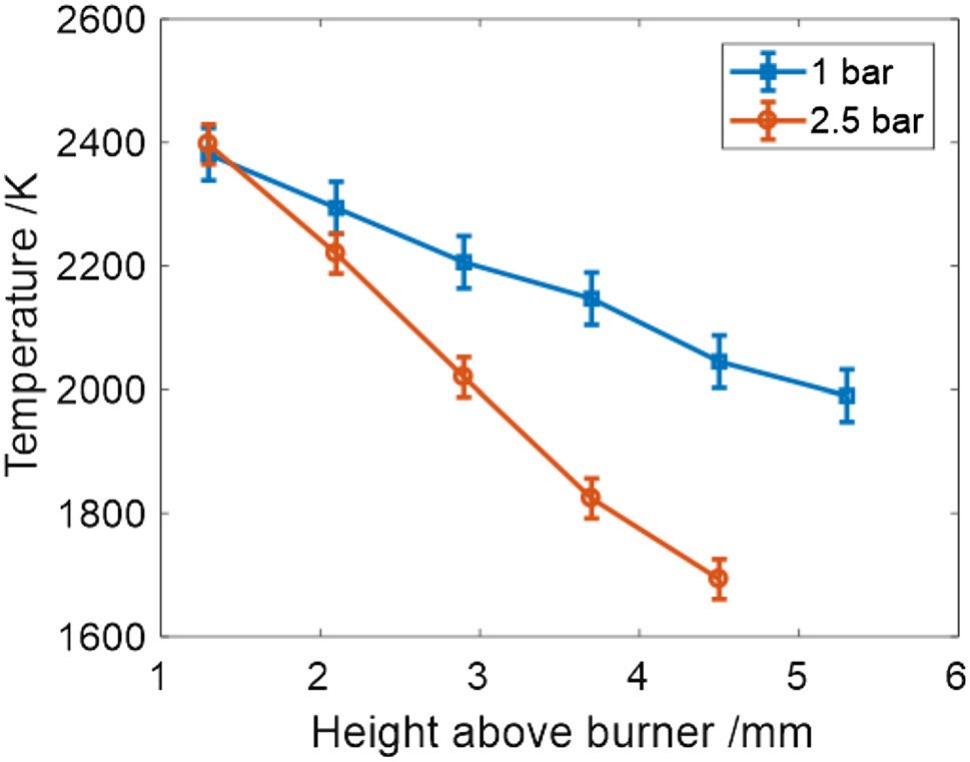
Temperatures at different HAB for flame pressures of 1 and 2.5 bar with 25:75 O_2_:N_2_ content in the pre-mixed methane air flame. Error bars are based on the “goodness of fit” uncertainty and uncertainties in *γ*/*m* and Λ

With a gas mixture having a O_2_:N_2_ molar ratio of 25:75, the temperature was measured as a function of height above burner HAB, at 1 and 2.5 bar. The variation in temperature with increasing HAB is shown in Fig. 10 for the two conditions of 1 and 2.5 bar total pressure.

### Concentration of NO

**Fig. 11:**
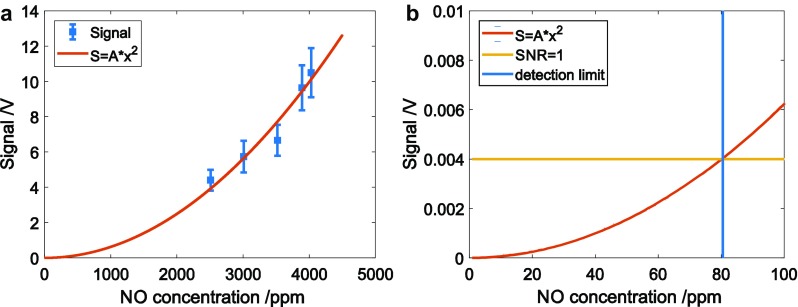
**a** Peak LITGS signal vs. simulated concentration of NO in the flames with different O_2_ concentration. The points follow a *S* = *A* × *x*^2^ curve. **b** Extrapolation of the fitted curve down to the point where the SNR = 1, and the resulting detection limit

The concentration of NO in the burnt gas for different O_2_ contents in the gas mixture was calculated to be in the range 2000–5000 ppm and the LITGS signal strength, *S*, plotted as a function of the calculated concentration, *N*_NO_. The result, for a flame at 1 bar, is shown in Fig. 11a and indicates that, as expected from the theory, $S \propto N_{{{\text{NO}}}}^{2}.$ The LITGS technique, therefore, appears to scale favourably for high-pressure measurements in the product gases of engines and gas turbines. In the case of engines, where peak pressures can reach 50–100 bar, this means that temperature measurements, using typical NO concentrations of up to 1000 ppm, are feasible with probe volumes of the order of cubic millimetres. Using averages over 50 single shots, a noise level of the order of 4 mV allows a minimum detection limit for a 5-s average measurement (10 Hz repetition rate of the laser) of ~ 80 ppm, as shown in Fig. 11b. Similar concentration levels can be detected using DFWM, although the strong inverse dependence of DFWM signal intensity on pressure precludes its use in high-pressure environments. However, DFWM was used to detect NO in a firing internal combustion engine, where concentrations of the order of 1000 ppm were present [13, 14].

## Conclusion

This work has investigated the use of simultaneous DFWM and LITGS signal generation for measurements in flames. The relative contribution of both signal generation processes using only the degenerate probe in a DFWM process have been illustrated by the time behaviour of the signals showing the transition from signals dominated by the coherent population grating at low pressures to those dominated by thermal gratings at higher pressures under unsaturated and saturated pump conditions. These results confirm previous studies made using a variable delay, pulsed probe to record the signals [18]. It has been shown that under suitable conditions it is possible to isolate each contribution, and thus, in principle, to use DFWM to monitor NO concentration and LITGS to obtain accurate and precise values of temperature in the same region of interest. The results presented here indicate that, under certain conditions, the presence of the DFWM probe to produce the DFWM signal will affect the intensity of the LITGS signal. Such “interference”, under these conditions would complicate the interpretation of LITGS signal intensities in terms of species concentration. This interference, however, would not affect the accuracy or precision of temperatures derived from the oscillation frequency of the signal. The intensity of the LITGS signal seems to be affected by factors that can be difficult to quantify, such as variation of gas dynamic parameters and of quenching rates as a function of temperature and pressure. In addition, the measurement of intensity is always prone to errors associated with fluctuations in the intensity or frequency of the excitation laser. The intensity of the DFWM signal can, however, provide information on relative concentrations as demonstrated in previous work to detect combustion-generated NO in an internal combustion engine [14].

The main conclusion is that accurate and precise measurements of temperature can be made using LITGS signals from NO in stable, laminar, pre-mixed CH_4_/O_2_/N_2_ flames at moderate pressures around 1 bar. As in previous studies, it is found that the precision improves with increasing pressure. The variation of flame temperature with oxygen content and position in the flame has been measured with good precision sufficient to detect fluctuations arising from flame flicker or other external perturbations. Thermometry using LITGS of NO in hydrocarbon/air flames has been shown also to be a potential method for measurements in a standardized flame system to provide a traceable standard for measurements in flames or at flame temperatures. Although a relatively large probe volume has been used in the present experiments at low pressures, the dependence of the signal on the square of the density suggests that higher spatial resolution would be possible for these measurements to obtain suitable signals in high-pressure combustors and engines. Turbulent combustion would present additional difficulties and mandate much smaller measurement volumes in order to avoid inclusion of regions of differing temperature. Temporal variations associated with turbulence could be addressed, in principle, by the use of high repetition rate laser systems. Such high-speed measurements have recently been demonstrated at rates up to 10 kHz [41].

## References

[1] Eckbreth AC (1996). Laser Diagnostics for Combustion Temperature and Species.

[2] Kohse-Höinghaus K, Jeffries JB (2002). Applied Combustion Diagnostics.

[3] Seitzman JM, Kychakoff G, Hanson RK (1985). Opt. Lett.

[4] Lee MP, McMillin BK, Hanson RK (1993). Appl. Opt.

[5] Roy S, Gord SJR, Patnaik AK (2010). Prog. Energy Combust. Sci.

[6] Kiefer J, Ewart P (2011). Prog. Energy Combust. Sci.

[7] Ewart P, O’Leary SV (1986). Opt. Lett.

[8] Dreier T, Rakestraw DJ (1990). Opt. Lett.

[9] Ewart P, Kaczmarek M (1991). Appl. Opt.

[10] Yip B, Danehy PM, Hanson RK (1992). Opt. Lett.

[11] Jefferies IP, Yates AJ, Ewart P, Castellucci E, Righini R, Foggi P (1993). Coherent Raman Spectroscopy—Applications and New Developments.

[12] R.L. Vander Wal, R.L. Farrow, D.J. Rakestraw, *24th Symposium (International) on Combustion* (The Combustion Institute, 1992) p. 1653

[13] Grant AJ, Ewart P, Stone CR (2002). Appl. Phys. B.

[14] Stevens R, Ewart P, Ma H, Stone CR (2007). Combust. Flame.

[15] Danehy PM, Friedman-Hill EJ, Lucht RP, Farrow RL (1993). Appl. Phys..

[16] Paul PH, Farrow RL, Danehy PM (1995). J. Opt. Soc. Am. B.

[17] Williams S, Rahn LA, Paul P, Forsman J, Zare RN (1994). Opt. Lett.

[18] Danehy PM, Paul PH, Farrow RL (1995). J. Opt. Soc. Am. B.

[19] Hubschmid W, Hemmerling B, Stampanoni-Panariello A (1995). J. Opt. Soc. Am. B.

[20] Cummings EB (1994). Opt. Lett.

[21] Cummings EB, Leyva IA, Hornung HG (1995). Appl. Opt.

[22] Stampanoni-Panariello A, Hemmerling B, Hubschmid W (1995). Phys. Rev.

[23] Stampanoni-Panariello DN, Kozlov PP, Radi B, Hemmerling (2005). Appl. Phys. B.

[24] Latzel H, Dreizler A, Dreier T, Heinze J, Dillmann M, Stricker W, Lloyd GM, Ewart P (1998). Appl. Phys. B.

[25] Stevens R, Ewart P (2004). Appl. Phys. B.

[26] Williams B, Edwards M, Stone R, Williams J, Ewart P (2014). Combust. Flame.

[27] Hell A, Förster FJ, Weigand B (2016). J. Raman Spectrosc.

[28] Sahlberg A, Hot D, Kiefer J, Aldén M, Li ZS (2016). Proc. Combust. Inst..

[29] Docquier N, Candel S (2002). Prog. Energy Combust. Sci.

[30] Williams TC, Schefer RW, Oefelein JC, Shaddix CR (2007). Rev. Sci. Instrum.

[31] Marble FE, Candel SM (1977). J. Sound Vib.

[32] Polifke W, Paschereit CO, Döbbeling K (2001). Int. J. Acoust. Vib.

[33] Morgan AS, Duran I (2016). Int. J. Spray Combust. Dyn.

[34] Ewart P (1985). Opt. Commun.

[35] Sun ZW, Li ZS, Li B, Aldén M, Ewart P (2010). Appl. Phys. B.

[36] Shirley JA, Hall RJ, Eckbreth AC (1980). Opt. Lett.

[37] Fantoni R, De Dominicis D, Georgi M, Williams RB (1996). Chem. Phys. Lett.

[38] W.G. Bessler, C. Schulz, V. Sick, J.W. Daily, in *Proceedings of the Third Joint Meeting of the U.S. Sections of The Combustion Institute*, Chicago, March 16–19, 2003, paper PI05

[39] Abrams RL, Lind RC (1978). Opt. Lett.

[40] S. Gordon, B.J. McBride, Computer Program for Calculation of Complex Chemical Equilibrium Compositions and Applications. https://www.grc.nasa.gov/www/CEAWeb/RP-1311.htm. Accessed 2017

[41] Förster FJ, Crua C, Davy M, Ewart P (2017). Exp. Fluids.

